# Supplementary material for: Adherence to Mediterranean Diet and Food Neophobia Occurrence in Children: A Study Carried out in Italy
Source: Nutrients. 2023 Dec 12;15(24):5078. doi: 10.3390/nu15245078 (PMC10746015; doi:10.3390/nu15245078)
Supplement: Supplementary file 1 [file nutrients-15-05078-s001.zip › nutrients-2727067-supplementary.pdf]

Supplementary Materials: Table S1 - The Questionnaire

Questions	Answers
**PERSONAL DATA**	
**Gender**	Male/Female
**Age (years)**	Age in years
**Weight**	kg
**Height**	cm
**School attended by the child**	Nursery/Primary school
**Ethnic group**	African/Asiatic/Caucasian/Eurasian/Hispanic
**Household income**	Up to 10.000 euros/Between 10.001 and 25.000 euros/Between 25.001 and 40.000 euros/Between 40.001 and more
**Parents employment**	Both parents employed/≤1 parent employed
**Children in family**	Number in units
**SECTION 1 (S1) - CHILD FOOD NEOPHOBIA SCALE (CFNS)**	
S1.1 The child constantly sampling new and different food<br>S1.2 The child doesn't trust new foods<br>S1.3 If the child does not know what a kind of food is, he does not try it<br>S1.4 The child likes food from different cultures<br>S1.5 Ethnic food looks to him/her too weird to eat<br>S1.6 During a party the child is willing to try new foods<br>S1.7 The child is afraid to eat things he has never had before<br>S1.8 The child is very particular about the food he/she eats<br>S1.9 The child will eat almost anything<br>S1.10 The child likes to try new ethnic restaurants	For each question, choose one of the following options:<br>Strongly disagree/Disagree/Somewhat disagree/Neither agree nor disagree/Somewhat agree/Agree/Strongly agree
**SECTION 2 (S2) - KIDMED TEST**	
S2.1 Takes a fruit or fruit juice every day<br>S2.2 Has a second fruit every day<br>S2.3 Has fresh or cooked vegetables regularly once a day<br>S2.4 Has fresh or cooked vegetables more than once a day<br>S2.5 Consumes fish regularly (at least 2–3 times per week)<br>S2.6 Goes more than once a week to a fast-food (hamburger) restaurant<br>S2.7 Likes pulses and eats them more than once a week<br>S2.8 Consumes pasta or rice almost every day (5 or more times per week)<br>S2.9 Has cereals or grains (bread, etc.) for breakfast<br>S2.10 Consumes nuts regularly (at least 2–3 times per week)<br>S2.11 Uses olive oil at home<br>S2.12 Skips breakfast<br>S2.13 Has a dairy product for breakfast (yoghurt, milk, etc.)<br>S2.14 Has commercially baked goods or pastries for breakfast<br>S2.15 Takes two yoghurts and/or some cheese (40 g) daily<br>S2.16 Takes sweets and candy several times every day	Yes/No

Supplementary Materials: Table S2 - Results of the KIDMED Test

		n	%
**Fruit or fruit juice every day**	Yes	229	79.5
	No	59	20.5
**Second fruit every day**	Yes	110	38.2
	No	178	61.8
**Fresh or cooked vegetables regularly once a day**	Yes	92	31.9
	No	196	68.1
**Fresh or cooked vegetables more than once a day**	Yes	77	26.7
	No	211	73.3
**Fish at least 2-3 times per week**	Yes	188	65.3
	No	100	34.7
**Fast-food more than once a week**	Yes	54	18.8
	No	234	81.2
**Legumes more than once a week**	Yes	133	46.2
	No	155	53.8
**Pasta or rice 5 or more times per week**	Yes	90	31.3
	No	198	68.7
**Cereals or grains (bread, etc.) for breakfast**	Yes	54	18.8
	No	234	81.2
**Nuts at least 2–3 times per week**	Yes	73	25.3
	No	215	74.7
**Olive oil as seasoning**	Yes	276	95.8
	No	12	4.2
**Skips breakfast**	Yes	34	11.8
	No	254	88.2
**Dairy product for breakfast (yoghurt, milk, etc.)**	Yes	264	91.7
	No	24	8.3
**Commercially baked goods or pastries for breakfast**	Yes	235	81.6
	No	53	18.4
**Two yoghurts and/or some cheese (40 g) daily**	Yes	171	59.4
	No	117	40.6
**Sweets and candy several times every day**	Yes	115	39.9
	No	173	60.1
